# Salt stress responsiveness of a wild cotton species (*Gossypium klotzschianum*) based on transcriptomic analysis

**DOI:** 10.1371/journal.pone.0178313

**Published:** 2017-05-26

**Authors:** Yangyang Wei, Yanchao Xu, Pu Lu, Xingxing Wang, Zhenqing Li, Xiaoyan Cai, Zhongli Zhou, Yuhong Wang, Zhenmei Zhang, Zhongxu Lin, Fang Liu, Kunbo Wang

**Affiliations:** 1 State Key Laboratory of Cotton Biology, / Institute of Cotton Research of Chinese Academy of Agricultural Science (ICR-CAAS), Anyang, Henan, China; 2 National Key Laboratory of Crop Genetic Improvement (Wuhan), Huazhong Agricultural University, Wuhan, Hubei, China; East Carolina University, UNITED STATES

## Abstract

Cotton is a pioneer of saline land crop, while salt stress still causes its growth inhibition and fiber production decrease. Phenotype identification showed better salt tolerance of a wild diploid cotton species *Gossypium klotzschianum*. To elucidate the salt-tolerant mechanisms in *G*. *klotzschianum*, we firstly detected the changes in hormones, H_2_O_2_ and glutathione (GSSH and GSH), then investigated the gene expression pattern of roots and leaves treated with 300 mM NaCl for 0, 3, 12, 48 h, and each time control by RNA-seq on the Illumina-Solexa platform. Physiological determination proved that the significant increase in hormone ABA at 48 h, while that in H_2_O_2_ was at 12 h, likewise, the GSH content decrease at 48 h and the GSSH content increase at 48 h, under salt stress. In total, 37,278 unigenes were identified from the transcriptome data, 8,312 and 6,732 differentially expressed genes (DEGs) were discovered to be involved in salt stress tolerance in roots and leaves, respectively. Gene function annotation and expression analysis elucidated hormone biosynthesis and signal transduction, reactive oxygen species (ROS), and salt overly sensitive (SOS) signal transduction related genes revealed the important roles of them in signal transmission, oxidation balance and ion homeostasis in response to salinity stress. This is a report which focuses on primary response to highly salty stress (upto 300 mM NaCl) in cotton using a wild diploid *Gossypium* species, broadening our understanding of the salt tolerance mechanism in cotton and laying a solid foundation of salt resistant for the genetic improvement of upland cotton with the resistance to salt stress.

## Introduction

Abiotic stresses such as salt excess (NaCl), drought, and temperature are among factors most limiting to plant productivity [1]. Plants have to evolve strategies to survive from abiotic and biotic stresses because they cannot escape from stress by moving to places that are more favorable. Therefore, the complete systems have been formed over the long course of evolution by plants to perceive, transduce, and respond to stresses at the molecular, cellular, and physiological levels. Salt stress is the most serious abiotic stress which threat to agriculture and environment in many parts of the world. It could destroy ion homeostasis, osmotic homeostasis, and lead to redox imbalances, further cause the ion toxicity, oxidative damage, and water-deficit, as a consequence of inhibition of photosynthesis, metabolic dysfunction, and damage to cellular structures within plant cells. In order to survive under such conditions, plants have developed the ability of perceiving and responding to these stresses rapidly via signal transduction pathways [2]. Although the molecular mechanisms are relatively complex comparing to the physiological and biochemical process, an increasing number of studies have focused on this field and have achieved a consensus in model plants such as *Arabidopsis* and rice [3]. The stress information was perceived by an unknown sensor in the plasma, while the signal with transmission result in the activation of the stress-activated transcription factors initiates transcriptional reprogramming. Eventually, some active proteins directly or indirectly participate in the maintenance of osmotic, ion, and redox balance [4, 5].

High concentrations of Na^+^ causes osmotic imbalance, membrane disorganization, reduction in growth, inhibition of cell division and expansion, which also leads to reduction in photosynthesis and production of reactive oxygen species[6, 7]. It is clear that various hormones, Ca^2+^-related and reactive oxygen species (ROS) signaling pathways play the key role in signaling transmission [8]. It is well known that abscisic acid (ABA), ethylene (ET), salicylic acid (SA) and jasmonates (JA) these four kinds of phytohormones play major roles in mediating plant defense response against pathogens and abiotic stresses [9–11]. Especially ABA, which is responsible for plant defense against abiotic stresses because environmental conditions such as drought, salinity, cold, heat stress and wounding are known to trigger increase in ABA levels [12, 13]. Briefly, salt overly sensitive pathway (SOS), which was known as a novel pathway linking the Ca^2+^ signaling in response to salt stress [14, 15], results in the exclusion of excess Na^+^ ions out of the cell via the plasma membrane Na^+^/H^+^ antiporter and helps in reinstating cellular ion homeostasis. A rapid increase in the rate of ROS production, known as ‘the oxidative burst’, occurs as a response to stress conditions, which always depends on the respiratory burst oxidase homolog protein (RBOH) [16]. High ROS levels not only result in oxidative damage to proteins, DNA, and lipids, but also act as signaling molecules to control various processes including pathogen defense, programmed cell death, and stomatal behavior [17–19]. To cope with the oxidative damage resulting from ROS, plants have developed a complex scavenging system, components of this system include enzymatic antioxidants (SOD, APX, PRX, GPX, CAT, GRX and TRX) and non-enzymatic scavengers (AsA, GSH, tocopherols, carotenoids and phenolic compounds) [20, 21]. Many signal transduction pathways involved ROS stress have been reported, such as abscisic acid (ABA) signaling pathway [22, 23], Ca^2+^ signaling pathway [24]. H_2_O_2_, one kind of mild ROS, also plays as the signal molecule in the antioxidation [25]. These signaling pathways work together to adaptive the salt stress through activating complex regulatory networks, which control global gene expression, protein modification and metabolite composition [26].

Cotton is a relatively salt tolerant species, but growth inhibition still occurs when the plant is exposed to saline stress. Germination and seedling growth are the two growing procedures particularly with salt sensitive [27, 28]. As such, in order to breed salt-tolerant cotton cultivars, researchers have focused on investigating the key molecular factors involved in the response to salt stress. Previous studies have shown that ectopic expression of cotton CBL-interacting protein kinase gene (*GhCIPK6*) and *SnRK2* could enhance abiotic stress tolerance [29, 30]. Furthermore, over-expressing the vacuolar location *AtNHX1* gene in cotton can also improve salt tolerance [31]. Evidence from transgenic plants has demonstrated the important role of transcription factors under salt stress in cotton. Such as *GhWRKY39-1* and *GhDREB1* enhance abiotic stress tolerance in transgenic plant [32, 33] and over-expression of the rice NAC gene, *SNAC1*, could also improve salt tolerance in transgenic cotton [34]. Overexpression ROS scavengers, such as *GhSOD1*, *GhCAT1*, and *GhMT3a*, showed the high salt stress tolerance in cotton [35, 36].

In addition, the completion of the *G*. *raimondii* [37, 38], *G*. *arboretum* [39], *G*. *hirsutum* [40, 41], and *G*. *barbadense* [42] whole genome sequencing offers a new model system for better understanding the adaptive mechanisms in extreme environments. Transcriptomic analysis provides detailed information about gene expression at the mRNA level and is widely used to screen candidate genes involved in stress responses. Recently, several transcriptome studies have contributed to our knowledge of the molecular regulatory pathways to salt stress tolerance and adaptation in cotton, such as miRNA networks by comprehensive analysis of two contrasting cotton genotypes under salt stress [43], such as the protein network of early salt stress responsiveness has also been revealed by iTRAQ in upland cotton [44]. However, major RNA-Seq studies on cultivated cotton have contributed to the knowledge of the mRNA regulatory pathways to salt stress tolerance [45–49], there still is insufficient knowledge of cotton salt tolerance mechanism. So it is necessary that the studies on non-cultivated cotton species, in order to supplement cotton salt tolerance mechanism. Two wild cotton species, *G*. *aridum* [50] and *G*. *davidsonii* [51] showed their tolerance to salt stress, whereas the responses were tested on the low NaCl concentration (200 mM) and the response knowledge of high NaCl concentration, e.g. 300 mM NaCl, needs to be understood. Furthermore, few studies took concentrations on the plant hormones response to salt stress in cotton. Hence, we designed the experiment using the wild diploid cotton species *G*. *klotzschianum* to decipher the mechanism of cotton’s rapid response to 300 mM NaCl salinity stress.

Here, a cotton D-genome diploid species, *G*. *klotzschianum*, was selected for transcriptome analysis to identify the regulation networks involved in responses to salinity stress. On account of its sister species *G*. *davidsonii* has already been proved salt tolerance by physiological and RNA-Seq analysis under mild stress [51], the same phenotype of *G*. *klotzschianum* and *G*. *davidsonii* in various concentration salt stresses demonstrated the salt tolerance of *G*. *klotzschianum*. Likewise, the contents of four kinds of phytohormones (ABA, ETH, GA, IAA), H_2_O_2_, AsA, and glutathione in leaves were measured to exam the response mechanism of high salinity tolerance in cotton. The transcriptome analysis of leaves and roots over 48 h (0, 3, 12, and 48) in response to 300 mM NaCl stress were designed to investigate regulatory network at early stage under severe salt stress. The study not only provides supplement data for cotton salt tolerance research but also lays a solid foundation on engineering breeding for salt resistance in cotton.

## Materials and methods

### Plant materials and salt stress treatments

The plant materials in the study were two diploid D genome wild cotton species *G*. *klotzschianum* (accession D_3k_-01) and *G*. *davidsonii* (accession D_3d_-01), which were grown in the greenhouses at the Institute of Cotton Research, Chinese Academy of Agricultural Sciences (ICR-CAAS) (Anyang, Henan, China) and also perennially maintained in the National Wild Cotton Nursery which locates at Sanya City, Hainan Island, China. The accession seeds were delinted and sterilized by using 1% of sodium hypochlorite for 15 min, followed by washing with sterile water for three times. Sterilized seeds were germinated at 28°C under long day conditions in a 16 h light/8 h dark cycle with a light intensity of 150μmol m^-2^s^-1^ on 17% water content sands. Three days after germination, the properly plants were potted in soil and placed in a growth room at 28°C, 60–70% relative humidity, a photoperiod of 16 h/8 h (day/night) and light intensity of 150μmol m^-2^s^-1^. Seedlings containing two simple leaves and one heart-shaped leaf were randomly selected to four groups, one group with three pots, then the plants were watered with 0, 200mM, 250mM, and 300 mM NaCl for 3 days, three biological replicates were prepared for each time point. The leaf and root samples of 300 mM NaCl treatment were harvested for varying durations (0 h, 3 h, 12 h, and 48 h) after exposing to salt stress, the control plants were also harvested at the same time point, three plants were collected and mixed, to minimize the effect of transcriptome unevenness among plants. For the treated leaves and roots, parts of them were used to RNA-seq, and parts of them were used to quantitative real-time PCR (qRT-PCR), parts of leaves were used to detect hormones (ABA, ETH, GA and IAA) and other biochemistry indexes (H_2_O_2_, GSH, GSSH and AsA). All the samples were frozen and stored in liquid nitrogen and stored at −70°C for further use (named GKLC0, GKLC3, GKLC12, GKLC48, GKLS3, GKLS12, GKLS48, GKRC0, GKRC3, GKRC12, GKRC48, GKRS3, GKRS12, GKRS48, where GK, L, R, C, and S correspond to *G*. *klotzschianum*, leaf, root, control and salt treatment, respectively).

### Measurement of the hormones, glutathione, H_2_O_2_, and AsA contents

The concentration of abscisic acid (ABA), ethylene (ETH), gibberellic acid (GA), and indole acetic acid (IAA) in leaves were determined using the ELISA method. ABA (CK-E00005P, DG, Beijing), ETH (CK-E00021P, DG, Beijing), GA (CK-E00001P, DG, Beijing), IAA (CK-E00015P, DG, Beijing), these four kind of kits were utilized for the determination of each hormone contents. 0.5 gram for each sample applied for the measurement ELISA (Infinite M1000, TECAN, Switzerland). Three replicated experiments (two-time technical repeats per biological replicate) were performed.

The contents of GSH (reduced glutathione) and GSSG (oxidized glutathione) were measured using a GSH and GSSG assay kit (Nanjing Jiancheng Bioengineering Institute). The reaction was initiated by the addition of H_2_O_2_. A series of enzymatic reactions were activated by GSH in the homogenate which subsequently led to the conversion of GSH (reduced glutathione) to oxidize glutathione (GSSG). The change in absorbance during the conversion of GSH to GSSG was recorded spectrophotometrically at 405 nm. Three replicated experiments (two-time technical repeats per biological replicate) were performed.

The content of H_2_O_2_ in leaves was assessed using a commercially available kit (Nanjing Jiancheng Bioengineering Institute). H_2_O_2_ bound with molybdenic acid to form a complex, which was measured at 405 nm and the content of H_2_O_2_ was then calculated. The content of AsA in leaves was assessed using a commercially available kit (Nanjing Jiancheng Bioengineering Institute), which was measured at 265 nm and the content of AsA was then calculated. Three replicated experiments (two-time technical repeats per biological replicate) were performed.

### RNA extraction, cDNA library construction, and RNA-Seq

Total RNA of each sample was extracted from the cotton samples according to the instruction manual of the TRlzol Reagent (Life technologies, California, USA). RNA integrity and concentration were checked using an Agilent 2100 Bioanalyzer (Agilent Technologies, Inc., Santa Clara, CA, USA). The mRNA was isolated by NEB Next Poly (A) mRNA Magnetic Isolation Module (NEB, E7490). The cDNA library was constructed following the manufacturer’s instructions of NEB Next Ultra RNA Library Prep Kit for Illumina (NEB, E7530) and NEB Next Multiplex Oligos for Illumina (NEB, E7500). In briefly, the enriched mRNA was fragmented into approximately 200nt RNA inserts, which were used to synthesize the first-strand cDNA and the second cDNA. The double-stranded cDNA were performed end-repair/dA-tail and adaptor ligation. The suitable fragments were isolated by Agencourt AMPure XP beads (Beckman Coulter, Inc.), and enriched by PCR amplification. Finally, the constructed cDNA libraries of the cotton were sequenced on a flow cell using an Illumina HiSeq™ 2500 sequencing platform.

### Mapping and sequence annotation

Low quality reads, such as only adaptor, unknown nucleotides>5%, or Q20 <20% (percentage of sequences with sequencing error rates <1%), were removed by per script. The clean reads that were filtered from the raw reads were mapped to cotton genome (*G*. *raimondii*; JGI v2.1) using Tophat2 software [52]. The aligned records from the aligners in BAM/SAM format were further examined to remove potential duplicate molecules. Gene expression levels were estimated using FPKM values (fragments per kilobase of exon per million fragments mapped) by the Cufflinks software [52]. Genes were compared against various protein databases by BLASTX, including the National Center for Biotechnology Information (NCBI) non-redundant protein (Nr) database, Swiss-Prot database with a cut-off E-value of 10^−5^. Furthermore, genes were searched against the NCBI non-redundant nucleotide sequence (Nt) database using BLASTn by a cut-off E-value of 10^−5^. Genes were retrieved based on the best BLAST hit (highest score) along with their protein functional annotation. To annotate the gene with gene ontology (GO) terms, the Nr BLAST results were imported into the Blast2GO program [53]. GO annotations for the genes were obtained by Blast2GO. This analysis mapped all of the annotated genes to GO terms in the database and counted the number of genes associated with each term. Perl script was then used to plot GO functional classification for the unigenes with a GO term hit to view the distribution of gene functions. The obtained annotation was enriched and refined using TopGo (R package). The gene sequences were also aligned to the Clusters of Orthologous Group (COG) database to predict and classify functions [54]. KEGG pathways were assigned to the assembled sequences by perl script.

### Identification of differential gene expression

DESeq and Q-value were employed and used to evaluate differential gene expression between control and treatment [55]. After that, gene abundance differences between those samples were calculated based on the ratio of the FPKM values. The false discovery rate (FDR) control method was used to identify the threshold of the P-value in multiple tests in order to compute the significance of the differences. Here, only gene with an absolute value of log2 ratio ≥ 2 and FDR significance score < 0.01 were used for subsequent analysis.

### Validation of RNA-Seq by qRT-PCR

Real-time RT-PCR (qRT-PCR) was performed on a new set of 3 replicates for each sample. A set of 38 genes was chosen randomly (S1 Table) from roots and leaves by dividing their expression levels at different time points with their observed FPKM. In addition, to further verify the correctness of the analysis results, nineteen SOS-, ABA- and ROS-related genes were selected and detected by qRT-PCR (S1 Table-Further Examination). Reverse transcription was conducted with the GoScriptTM Reverse Transcription System (Promega, Madison, USA). NCBI primer-BLAST was employed to design specific primers for the chosen genes. Using Fast Start Universal SYBR Green Master (Rox) mix (Roche), real-time PCR was conducted in a 7500 fast Real-Time PCR system (Applied Biosystems), and the results were analysed via the ΔΔCt method *Gractin7* (F: ATCCTCCGTCTTGACCTTG, R: TGTCCGTCAGGCAACTCAT) gene was used as a control. Each reaction was carried out in a final volume of 20 μL, containing 10 μL of SYBR Green PCR master mix, 0.5 μL of each gene-specific primer and 2 μL of diluted cDNA. The PCR thermal cycling conditions were as follows: 95°C for 10 min; 40 cycles of 95°C for 5 s, 60°C for 30 s and 72°C for 30 s. Data were collected during the extension step: 95°C for 15 s, 60°C for 1 min, 95°C for 30 s and 60°C for 15 s. Three biological replicates were performed, and three technical replicates were designed per cDNA sample.

## Results

### Phenotypic and biochemistry responses to salt stress in *G*. *klotzschianum*

**Fig 1:**
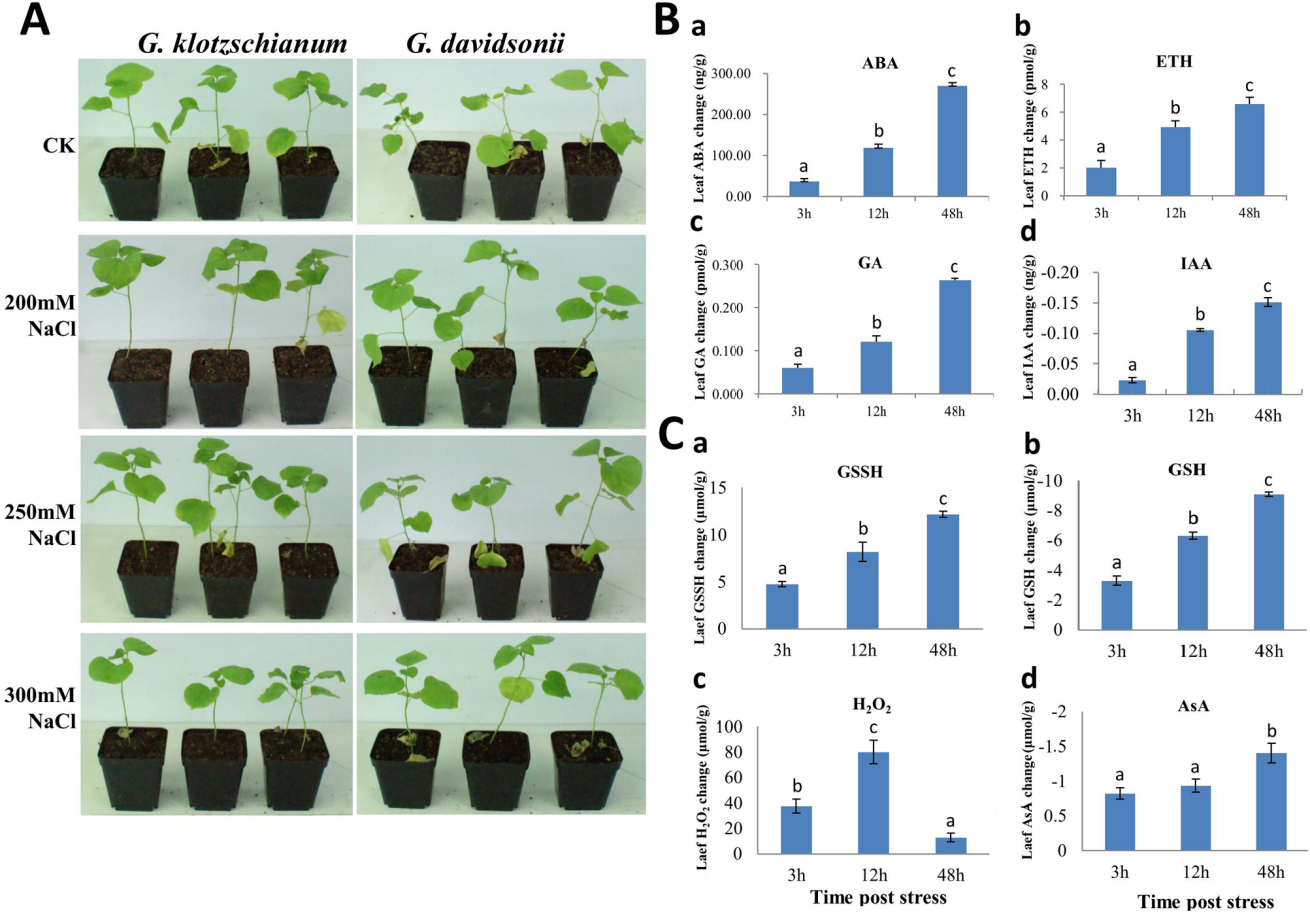
Phenotypic, biochemistry analysis of *G*. *klotzschianum* in response to salt stress. **A:** Phenotype of *G*. *klotzschianum* and *G*. *davidsonii* post salt stress (200 mM, 250 mM, 300 mM NaCl) for three days. **B a-d:** the changes of ABA, ETH, GA, and IAA concentration at different times post 300 mM NaCl stress. **C a-d:** the changes of GSSH, GSH, H_2_O_2_, and AsA concentration at different times post 300 mM NaCl stress. *G*. *klotzschianum* seedlings grown under normal conditions were used as controls. Three biological replicates were used. Multiple comparisons were performed with significant difference in different letter at P < 0.05 level; Error bars represent SD.

*G*. *klotzschianum* was considered as sister species of *G*. *davidsonii* in *Gossypum* taxonomy and *G*. *davidsonii* has been proved with better salt-tolerances by the previous study [51]. Our results showed that *G*. *klotzschianum* owned the same phenotypic feature to *G*. *davidsonii* after post in various NaCl concentrations of salt stress for 3 days (Fig 1A). The old leaves of 200 mM and 250 mM NaCl treatments became yellow comparing to control, and dropped down after post at the 300 mM NaCl after 3 days posted salt stress. Furthermore, the contents of ABA were significantly increased at three time points in leaves with 300 mM NaCl treatment as compared to control (Fig 1B-a), and got the maximum value at 48h. In addition the contents of ETH and GA also kept increases after post at 300 mM NaCl salt stress at three time points, with the same tendency of ABA (Fig 1B b-c), but the contents of IAA kept decreases lower than control after post at salt stress at three time points, which revealed the completely opposite tendency with ABA (Fig 1B d), all these results suggesting that the different roles of hormones in responding to salt stress. A significant increase in H_2_O_2_ was observed at 3, 12, and 48 h compared to each of their corresponding controls (Fig 1C a), which showed a statistically significant increase at 12 h. Likewise the GSSH contents were keeping increase under salt stress and maximizing at 48 h, while the GSH contents showed a completely opposite pattern compared with GSSH at the same time point (Fig 1C b-c). Further, the AsA contents remain unchanged within 3 h and 12 h salt treatments, while increases 48 h (Fig 1C d). These findings suggested that these time points might show a substantial change in the expression of salt responsive genes, especially ROS/hormone-related genes. Therefore, RNA-seq was followed to profile the gene expression at each time point to deeply understand the salt stress response in cotton.

### Processing and mapping of Illumina reads

Here, we used high-throughput RNA sequencing (RNA-seq) to investigate the changes in gene expression of the diploid D-genome species *G*. *klotzschianum* at 300 mM NaCl concentration. Three plants were collected and mixed with each sample in this study. In total 14 sequencing libraries were constructed from both the roots and leaves at 0, 3, 12 and 48 h post salt stress and control conditions. All the samples were sequenced by using an Illumina HiSeq™ 2500 sequencing platform. On average of 61.5 million raw reads for the 14 libraries were obtained (Table 1). After the process of adaptor deletion, junk filtering and low copy filtering, > 95% of the sequences were confirmed as clean data. Then the clean reads were mapped to cotton whole genome (*G*. *raimondii*) using Tophat2 software [52], and 64.39–71.52% of the total reads were mapped to the reference genome, and the unique mapped reads were 62.49%-69.02%. The mapped sequences were assembled with Cufflinks software guided by a reference annotation from JGI Genomes (*G*. *raimondii* 2.1) [38]. The RNA-Seq assays revealed that a total of 37,278 unigenes and 797 novel genes were found in our data. Among them, the unigenes (18,855, 50.83%) with length exceeds 1000bp showed the more enrichment than the number of unigene (15,591, 42.03%) with the length exceeds 300bp and less-than1000bp.

**Table 1:** Summary of RNA-Seq results and their matches in the *G*. *raimondii* genome.

Samples	Raw Reads(M)	Clean Reads(M)	GC Content	≥Q30	Mapped Reads(M)	Uniq Mapped Reads(M)	Multiple Mapped Reads(M)
**GKLC0**	71.6	71.4	44.73%	96.42%	71.52%	69.02%	2.49%
**GKLC3**	60.8	60.6	44.95%	96.33%	71.28%	68.77%	2.51%
**GKLS3**	53.8	53.6	44.41%	96.46%	69.96%	68.07%	1.89%
**GKLC12**	51.7	51.6	44.98%	94.24%	66.74%	64.05%	2.68%
**GKLS12**	58.5	58.3	44.92%	94.12%	65.97%	62.82%	3.15%
**GKLC48**	48.5	48.4	44.23%	94.21%	66.96%	64.97%	1.99%
**GKLS48**	63.0	60.2	44.50%	85.06%	64.39%	62.49%	1.90%
**GKRC0**	66.6	66.4	44.26%	96.29%	70.72%	67.91%	2.82%
**GKRC3**	49.1	49.0	44.62%	96.05%	69.45%	66.05%	3.41%
**GKRS3**	77.7	77.5	44.43%	95.85%	69.19%	66.51%	2.69%
**GKRC12**	72.3	72.1	44.50%	94.32%	66.67%	63.55%	3.12%
**GKRS12**	70.6	70.3	44.19%	95.85%	67.25%	65.14%	2.10%
**GKRC48**	60.8	60.7	44.27%	94.24%	65.72%	63.45%	2.27%
**GKRS48**	55.6	55.4	44.08%	94.62%	66.73%	65.08%	1.65%
**Average**	61.5	61.1	44.51%	94.58%	68.04%	65.56%	2.48%

### Exploration of DEGs in roots and leaves in response to salt stress

**Fig 2:**
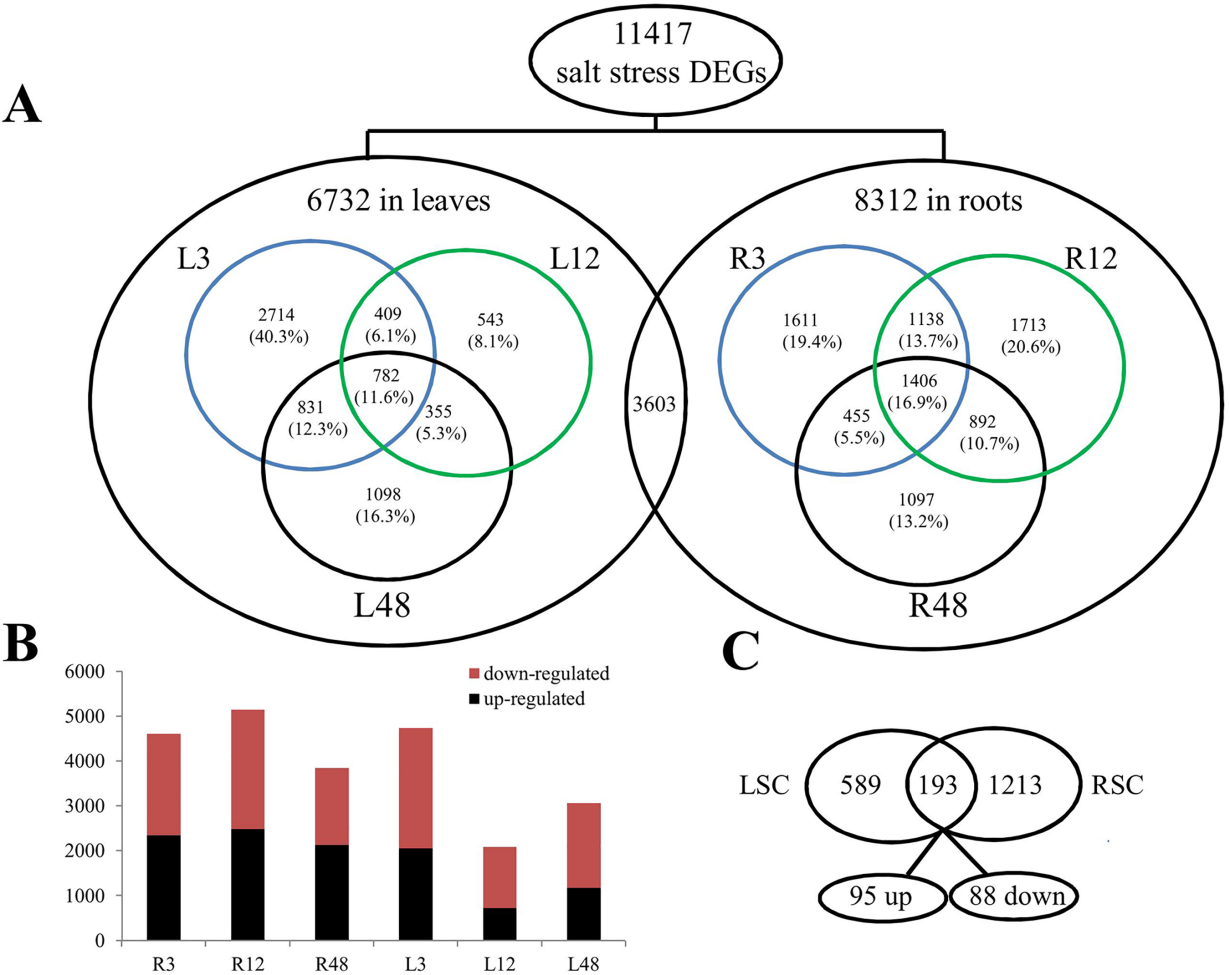
Summary of DEGs in leaves and roots of *G*. *klotzschianum* upon salt stress. **A:** Number of DEGs by salt stress under different time points (L3 GKLS3 vs. GKLC3, L12 GKLS12 vs. GKLC12, L48 GKLS48 vs. GKLC48, R3 GKRS3 vs. GKRC3, R12 GKRS12 vs. GKRC12, R48 GKRS48 vs. GKRC48), in leaves and roots. **B:** Comparion of up-/down-regulated genes in roots and leaves. **C:** Number of regulated genes between different conditions (LSC LS vs. LC, RSC RS vs. RC).

As the first step in the characterization of *G*. *klotzschianum* transcriptional responses to salt stress, we carried out the identification of the unigenes with expression level significantly changed upon NaCl-treatments. The transcript abundance of each gene was estimated by FPKM value. DESeq and Q-value were employed and used to evaluate differential gene expression pattern. A cutoff P-value < 0.01 adjusted by false discovery rate (FDR) and fold change ≥ 2 was used for identifying differentially expressed genes. A total of 11417 genes showed differential expression under salt stress conditions (L3 GKLS3 vs. GKLC3, L12 GKLS12 vs. GKLC12, L48 GKLS48 vs. GKLC48, R3 GKRS3 vs. GKRC3, R12 GKRS12 vs. GKRC12, R48 GKRS48 vs. GKRC48). In total, respectively, 8,312 and 6,732 DEGs were found in roots and leaves in response to salt stress. To identify candidate genes that respond to salt stress in *G*. *klotzschianum*, we adapted the DEGs identified by comparing the gene expression levels under salt stress vs. control conditions at the same time points. Meanwhile, 3,603 DEGs were commonly identified in the two tissue types, which highlights that 1,406 and 782 common genes with different expressions at three time points in roots (RSC RS vs. RC) and leaves (LSC LS vs. LC), respectively. The distribution of these genes is shown in a Venn diagram (Fig 2A). The results revealed that the induction of severe NaCl concentration would be more sensitive than mild NaCl concentration. Interestingly, there were several more DEGs in the roots than the leaves, and the up-regulated DEGs and down-regulated were with roughly the same number in roots, while, the down-regulated genes were more than the up-regulated genes in leaves in response to the salt stress (Fig 2B), which showed the significant inhibition of genes expression in leaves under salt stress. Likewise, 193 common genes were different expressed in the two organs, within them there were 95 genes up-regulated and 88 ones down-regulated (Fig 2C). These results are also in line with a transcriptionally higher responsivity of the roots system when compared to leaves’, as previously reported, e.g., in the case of two kinds of cotton [50, 51], which indicates tissue-specific responses to salt stress in *G*. *klotzschianum*.

Differentially regulated genes were also identified by comparing both of adjacent stages and every stage to 0h. “LC3/LC0” indicates a comparison between the gene expression in the GKLC3 library with that in the GKLC0 library, and the same describes the other labels (S2 Table). Interestingly, the up-regulated genes outnumbered the down-regulated genes between two time points under the same treatment.

**Fig 3:**
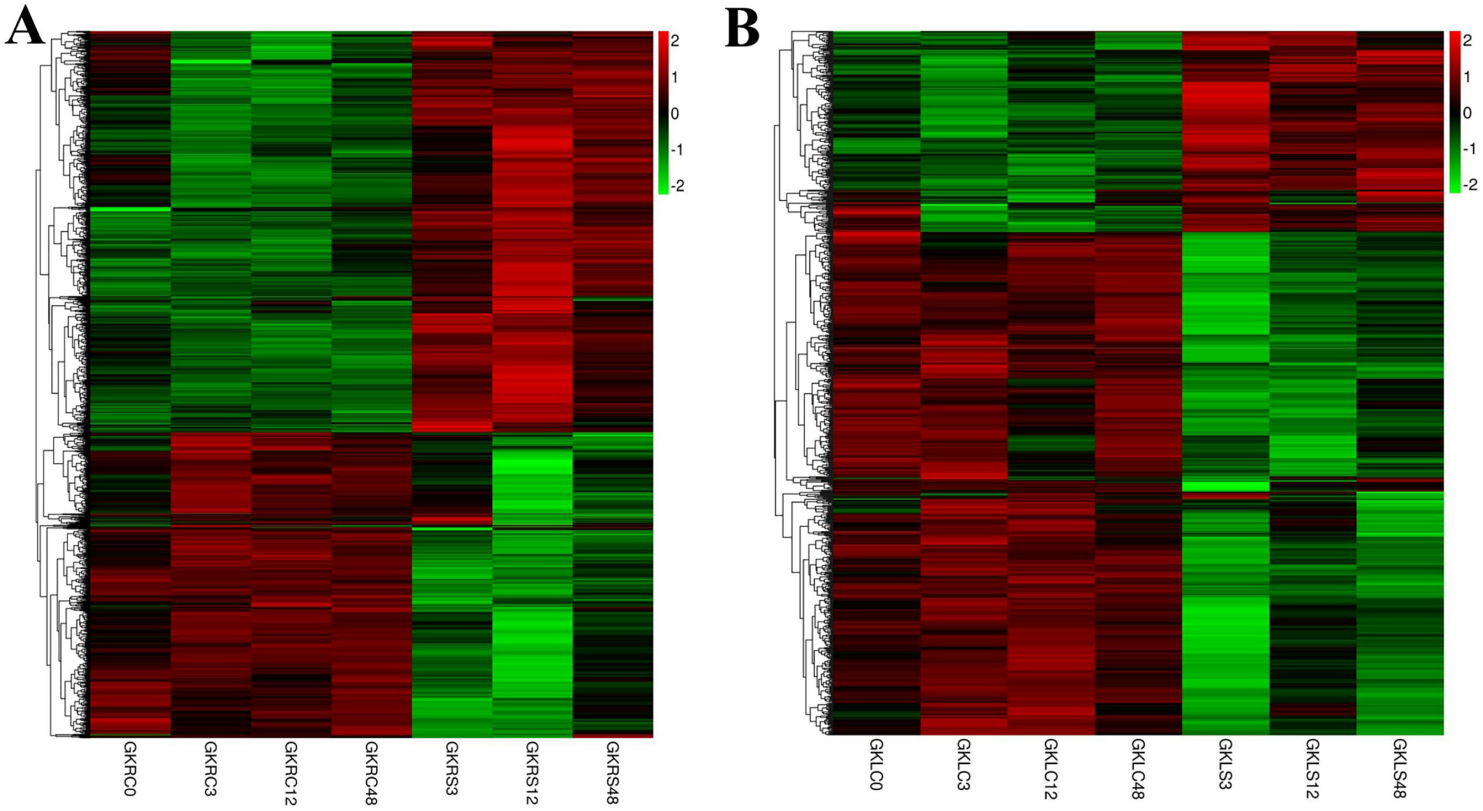
**A:** A heat map of 1406 differentially expressed genes from seven root samples. **B:** A heat map of 782 differentially expressed genes from seven leaf samples.

A cluster analysis of the common different expression genes at three time points in roots (1406) and leaves (782), respectively, resulted a dynamic change of gene expression by a heat map (Fig 3A and 3B), which implied the inhabitation of severe salt concentration in cotton. With comparing the 1406 and 782 DEGs in common at three time points in leaves (LSC LS vs. LC) and roots (RSC RS vs. RC), 193 common DEGs expressed in the two organs at three time points in total (S3 Table). We further observed a general conservation of expression patterns by the heat-map of these genes (S1 Fig), while 183 of the genes were regulated in the same way at all three time points. Among them, nine genes involved in ABA signal pathway were significant up-regulated, include two biosynthesis genes *NCED3* and seven signal transmission genes (six *PP2Cs* and one *ABF*). The results revealed that ABA plays the important role in cotton under salt stress.

**Fig 4:**
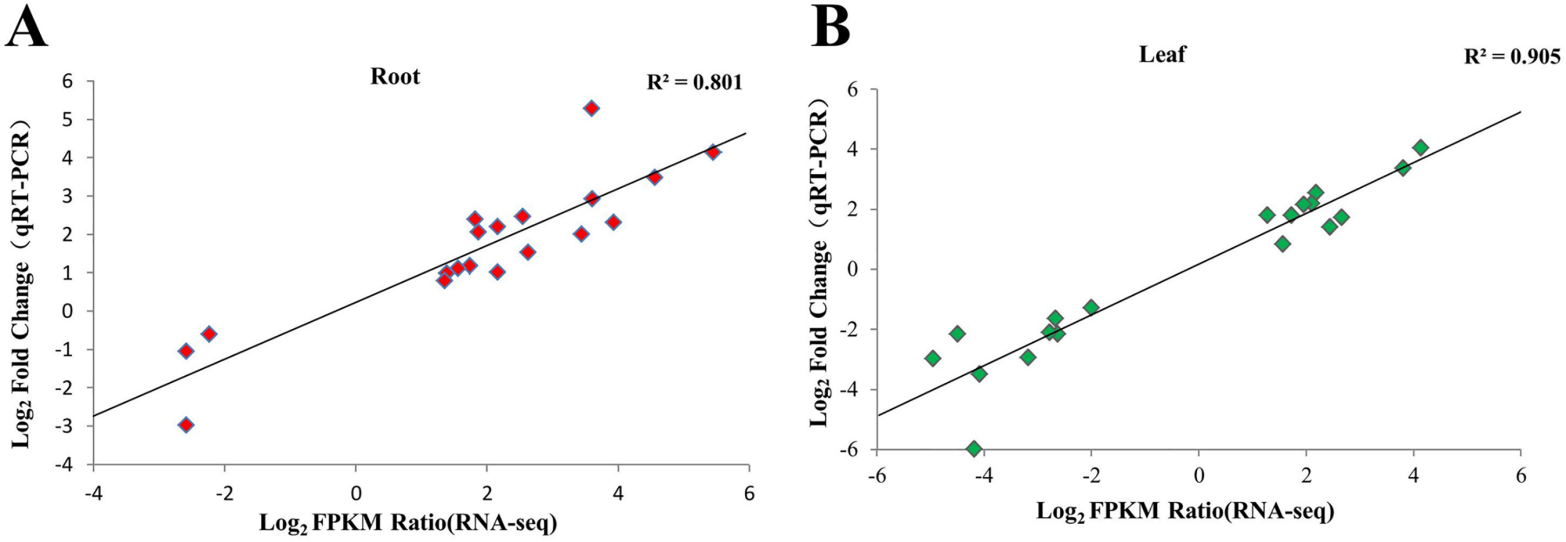
qRT-PCR validation of transcript levels evaluated by RNA-Seq in roots and leaves of *G*. *klotzschianum* under 300 mM NaCl stress conditions. **(A, B**) represent the correlation of the fold change analyzed by RNA-Seq platform (x-axis) with data obtained using real time PCR (y-axis) in roots and leaves of *G*. *klotzschianum*. 19 independent genes for each organ were randomly selected, three biological replicates were used from each sample at three time points post salt stress.

In order to validate the differential expression analysis by RNA-seq, 38 genes were chosen randomly from roots and leaves to confirm the veracity of RNA-seq data (S1 Table), including 19 genes for roots and 19 genes for leaves, respectively. Real-time PCR was performed on the same RNA pools that had been previously used for the next-generation sequencing. To corroborate the expression levels measured by RNA-Seq, the ratio of expression levels between salt stressed tissues and controls using RNA-Seq was compared to the ratio of expression as measured by qRT-PCR. A good correlation between RNA-Seq and real-time PCR results (coefficient of determination R^2^ = 0.801 and 0.905) indicates the reliability of RNA-Seq quantification of gene expression (Fig 4). The validation experiments support the accurateness of the relative values provided by the RNA-Seq analysis.

### GO and KEGG analysis of the DEGs

**Fig 5:**
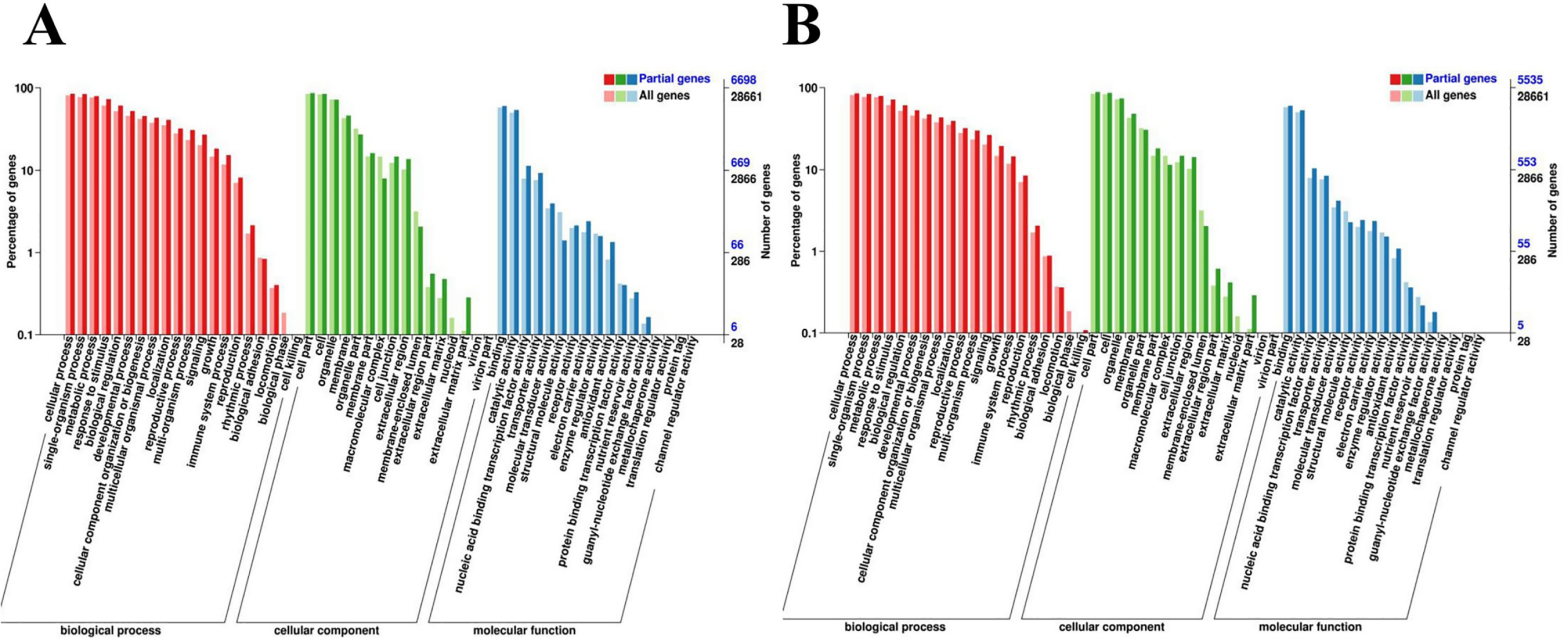
**A:** GO functional classification of DEGs in roots. **B:** GO functional classification of DEGs in leaves.

To understand DEGs’ function, GO category and KEGG pathway enrichment analysis were performed using P-value of 0.01 adjusted by false discovery rate (FDR) as the cutoff. 6,698 (80.58%) and 5,535 (82.22%) of the DEGs in roots and leaves, respectively, were annotated to GO terms. Further GO category enrichment analysis was performed using these DEGs. Respectively, 6,698 and 5,535 DEGs in roots and leaves were classified into 51 and 51 GO terms based on biological process, cellular component and molecular function (Fig 5A and 5B). In the molecular function, as comparing with the background genes, DEGs involved with antioxidant activity (90, 38.30%) nucleic acid binding transcription factor activity (763, 33.54%) were significant enriched in roots, followed by two GO terms, electron carrier activity (161, 31.76%) and transporter activity (623, 28.53%). Likewise, the genes sorted to the two GO terms electron carrier activity (130, 25.64%) and guanyl-nucleotide exchange factor activity (10, 25.64%) were revealed most different expression as comparing with the background genes in leaves, followed with antioxidant activity (60, 25.53%) and nucleic acid binding transcription factor activity (575, 25,27%). The results demonstrated the important role of the antioxidation under salt stress, which was consistent with the high level of glutathione (Fig 1C). Furthermore, for the number of DEGs, binding (4,072; 3,377) and catalytic activity (3,648; 2,961) showed the significantly enrichment in response to salt stress. For the biological process, genes involved with cell killing (5, 50%) and signaling (1825, 31.39%) were most different expression comparing with background genes in roots under salt stress. While in leaves, cell killing (6, 60%) and growth (1080, 25.63%) showed the most enriched, signaling term was at the third level (1474, 25.35%).

Further GO category enrichment analysis was performed using common DEGs. In our data, 1,191 (84.71%) and 680 (86.96) of the common DEGs in roots and leaves were classified into 44 and 46 GO terms based on biological process, cellular component and molecular function. For the molecular function, most DEGs were sorted into binding (724 genes, 51.49% of the 1406 DEGs; 411 genes, 52.55% of the 782 DEGs) and catalytic activity (684, 48.65%; 355, 45.40%). The GO term of response to stimulus in the biological process section, was significant enriched with the number of 941 genes, 66.93% of the 1406 DEGs, and 510 genes, 65.22% of the 782 DEGs, respectively. Furthermore, in order to understand the dynamic expression patterns of DEGs under salt stress, several key terms were investigated (Table 2). The results revealed that mass of DEGs enriched in the “response to stress”, “ion transport”, “response to hormone” along with “response to oxidative stress” these four terms at three time points in roots and leaves. Moreover, according to the number of DEGs in the categories of “cation transport”, “inorganic anion transport” and “monovalent inorganic cation transport”, ion stress was not in significantly different expression in leaves, only 2 common DEGs in the category of “cation transport” in leaves, whereas 7, 3, and 2 common DEGs in roots. The results are consistent with previous studies which showed that the salt stress would induce osmotic stress, ion stress, and ROS stress in plants [43, 49].

**Table 2:** GO terms related to stimulus or stress of DEGs.

GO Term	R3	R12	R48	RSC	L3	L12	L48	LSC
**response to osmotic stress**	92	91	69	29	63	35	53	9
**response to oxidative stress**	126	140	120	46	101	56	85	18
**response to starvation**	14	16	9	4	14	4	5	0
**response to salt stress**	473	505	392	253	424	233	320	129
**response to water deprivation**	511	536	424	185	461	208	286	80
**cation transport**	33	34	32	7	27	10	24	2
**inorganic anion transport**	9	9	6	3	11	1	5	0
**monovalent inorganic cation transport**	5	5	4	2	4	1	1	0
**metal ion transport**	134	127	117	52	132	68	87	26
**nitrate transport**	178	182	186	75	151	77	127	29
**response to abscisic acid stimulus**	31	36	20	12	31	18	17	7
**response to ethylene stimulus**	22	20	19	9	16	5	9	2
**response to hormone stimulus**	6	4	3	0	6	2	4	0
**response to jasmonic acid stimulus**	9	9	8	4	4	2	2	2
**response to salicylic acid stimulus**	9	10	7	5	8	5	6	4
**response to chitin**	398	462	291	121	338	134	227	32
**response to sucrose stimulus**	5	5	3	2	4	3	2	1

**Fig 6:**
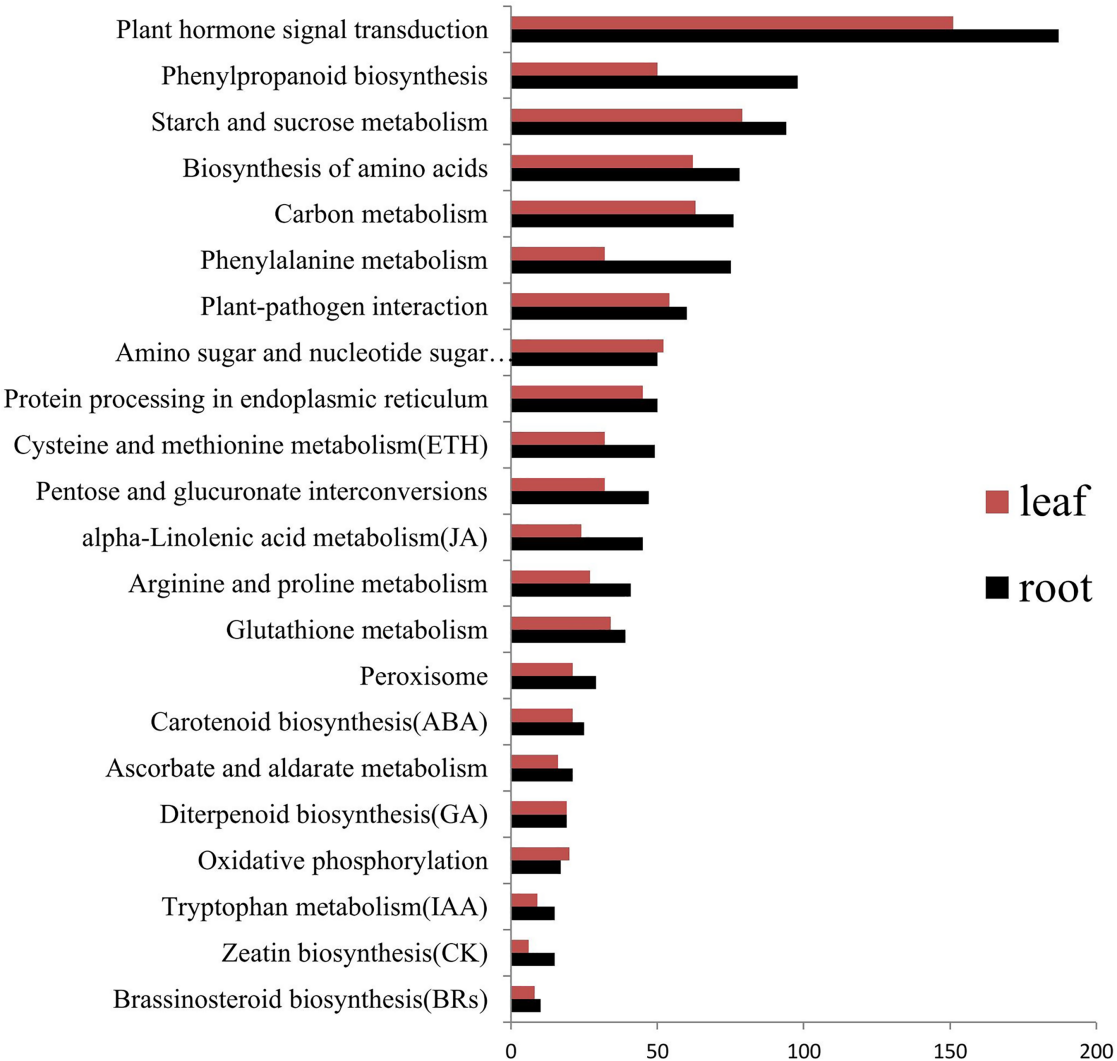
Hormones and ROS-related pathways under salt stress.

For the KEGG pathway enrichment analysis, 1452 (17.47%) and 1270 (18.87%) of the DEGs in roots and leaves, respectively, were annotated to KEGG pathway. A set of these DEGs were mapped onto KEGG pathways in *Arabidopsis thaliana* and *Oryza sativa*, highlighting the involvement of several hormone and ROS-related pathways in cotton (Fig 6). The plant hormone signal transduction pathway showed the most significant enrichment in roots (187, 12.88%) and leaves (151, 11.89%). Likewise, the biosynthetic pathways of seven hormones were also enriched in the DEGs (“carotenoid biosynthesis”, “zeatin biosynthesis”, “diterpenoid biosynthesis”, “brassinosteroid biosynthesis”, “cysteine and methionine metabolism”, “alpha-linolenic acid metabolism”, and “tryptophan metabolism”). Our data were in line with the previous studies that plant hormones play crucial roles in a diverse set of developmental processes, as well as in the response to biotic and abiotic stress [56–58]. Further, glutathione metabolism, peroxisome, ascorbate and aldarate metabolism, and oxidative phosphorylation of the four ROS-related pathways were also remarkably different expressed in roots and leaves.

In order to comprehend the dynamic change of gene expression among the two organs, 117, 120, 118, 119, 102, and 115 pathways were individual categorized in R3, R12, R48, L3, L12, and L48, respectively (S4 Table). Based on the ranks of the top 30 pathways from R3, the biosynthetic pathways of seven hormones were enriched in the DEGs (“carotenoid biosynthesis”, “zeatin biosynthesis”, “diterpenoid biosynthesis”, “brassinosteroid biosynthesis”, “cysteine and methionine metabolism”, “alpha-linolenic acid metabolism”, and “tryptophan metabolism”), as well as one pathway for “plant hormone signal transduction”. Interestingly, these eight pathways showed the most enrichment at 3h after post at salt stress, especially in roots. Likewise, we observed that the DEG numbers of hormone synthetic pathways showed the decrease with the duration exposure in at salt stress. The results were consistent with the previous transcriptome researches [59, 60]. Meanwhile, photosynthesis-antenna proteins and photosynthesis from these two photosynthesis-related pathways were observed in decrease at three time points in leaves, suggesting the severe damage of photosynthesis caused by salt stress. The results were implying the rapid response of plant hormone under salt stress.

Analyses on both the GO and KEGG enrichments demonstrated that the genes related to ion transport, ROS, hormones and signal transmission were significantly affected during the early stage of salt stress in cotton. Hence, we further explored that DEGs were involved in the metabolism of ROS and hormones, along with the signal transduction pathways systematically.

### DEGs involved in hormone biosynthesis and hormone signal transduction

**Fig 7:**
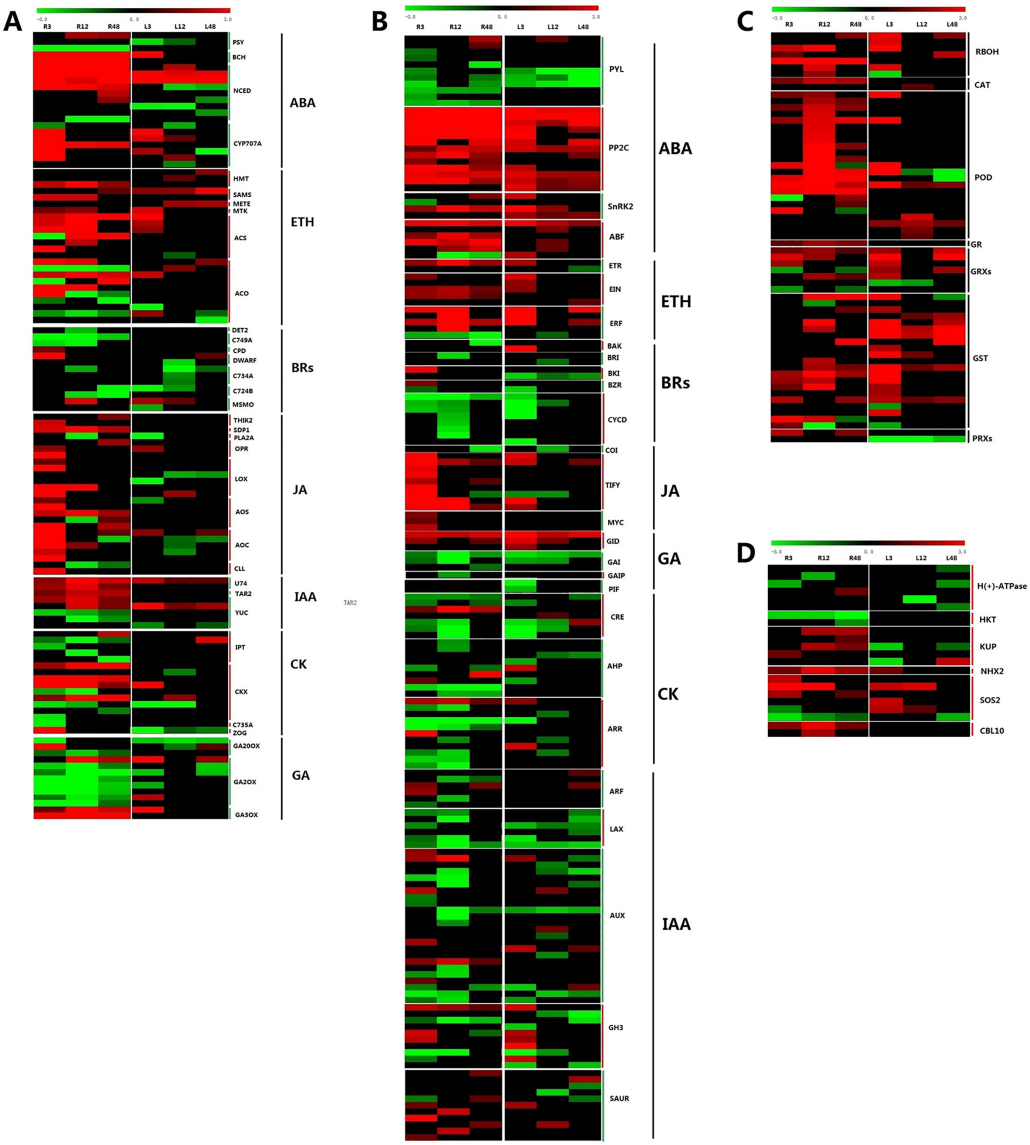
Expression profiling of hormone synthesis (**A**), hormone transduction (**B**), ROS (**C**), ion homeostasis (**D**) related genes.

Plant could perceive and respond to stresses rapidly via signal transduction pathways mediated by stress hormones [10, 11]. Hormones, in particular ABA along with cytokinins and ethylene, have been implicated in the root–shoot signaling, the long distance signaling may be mediated particularly via ABA as well as ROS [61]. To examine systematically the effect of salt stress on the pathways of hormone biosynthesis and plant hormone signal transduction, the seven hormones related KEGG pathways (biosynthesis of ABA, ethylene, BRs, JA, auxin, cytokinin, and GA) and plant hormone signal transduction pathway, were further examined manually (Fig 7A, S5 Table).

Under the salt stress, three phytoene synthase genes (*PSY*), two beta-carotene hydroxylase genes (*BCH*), nine 9-cis-epoxycarotenoid dioxygenase genes (*NCED*), and seven abscisic acid 8 hydroxylase genes (*CYP707A*) were significant different expression in the ABA biosynthetic pathway. Furthermore, most of *NCED* genes, an important biosynthetic gene which has been proved the important function on ABA biosynthesis [62], continuous increased at three time points in roots, as well as two NCED genes (*Gorai*.*002G038100*; *Gorai*.*013G177100*) were completely activated in two tissues. By contrast, the *CYP707A* genes, which were the key ABA catabolic genes, maintained higher expression at early stage under salt stress, while keep unchanged or down-regulated after exposure in salt stress 48h. Twenty-four genes involved in the ethylene biosynthetic pathway were detected as DEGs. Among them, two S-adenosylmethionine synthase (*SAM*) genes (*Gorai*.*008G135900*; *Gorai*.*005G071300*), precursor synthesis, were continuously upregulated. Meanwhile all seven 1-aminocyclopropane-1-carboxylate (*ACC*) synthase genes (*ACS*) were significantly induced, while half of ten 1-aminocyclopropane-1-carboxylate oxidase genes (*ACO*) were induced, and other five were inhibited gradually. These results showed that the key regulatory components of the biosynthetic pathways of two stress hormones, ABA and ethylene, were changed significantly during cotton’s response to salt stress (Fig 1B).

Many studies reported that BRs and JA play essential roles in plant responses to abiotic stress. The expression of thirteen genes involved in BRs biosynthetic pathways changed under salt stress (Fig 7A). Ten cytochrome P450 (*CYP*) genes participating in BRs biosynthesis in cotton, two *CYP749A22* (*Gorai*.*012G185300*; *Gorai*.*001G217300*) continuous expression in roots, while *CYP90A1* was only up-regulated before 12 h treatment in roots, and one of two *CYP85A* was activated. Three *CYP734A1* genes and two *CYP724B1* genes significant decreased. Only one genes encoding steroid 5-alpha-reductase (*DET2*), a major rate-limiting enzyme in BRs biosynthesis, showed decrease to more than 2-fold expression levels at 12h treatment in roots. Twenty-seven DEGs were identified involved JA biosynthesis pathway. And the expression of five allene oxide synthase (*AOS*) genes, which are the first enzyme in the branch pathway leading to JA biosynthesis, were significant increase after 3h treatment. These findings indicated that BRs and JA also actively participate in the stress response of cotton.

In addition, 14, 16, and 8 EDGs were also found in the biosynthetic pathway of GA, cytokinin, and auxin, which were widely considered to participate in regulating plant development. Interestingly, many of their genes showed increased transcript abundances in response to salt (Fig 7A).

In *Arabidopsis*, PP2C family genes were demonstrated to regulate ABA signaling pathways negatively by opposing the action of particular protein kinases and eight *PP2Cs* were characterized as key factors in ABA signaling transduction, otherwise *PP2Cs* could inactivate *SnRK2* via dephosphorylation, and this inactivation is inhibited by *PYR/PYL/RCRA*, ABA receptors, in an ABA-dependent manner [63–66]. In our data, thirty-four DEGs were participated in ABA signaling pathway and nine of eleven *PYLs* including *PYL1*, *4* and *8* were significantly down-regulated, especially with 3 members of the *PYL4* continuously down-regulated at both two organs under salt stress (Fig 7B, S6 Table). Fourteen *PP2Cs* were remarkably up-regulated, even six *PP2Cs* were de novo induced at the two tissues. Similarly, three of four *SnRK2s* were induced, the other one *SnRK2* was repressed at 3h time point in roots. Likewise, five of six *ABFs* expression increased, another one just decreased in roots at 12 h and 48 h under salt stress. These results were concord with early report [67], which showed the important role of ABA in the mechanism of salt tolerance in cotton. For ethylene signal pathway, one of two *ETRs* and five *EIN3s* were significant induced, while another *ETR* just decreased at 48 h in leaves under salt stress. Five *ERFs* were found to be changed by NaCl treatments, and most of them were up regulated. As for BRs and JA signal pathways, 16 and 19 genes were detected under salt stress (Fig 7B). Among DEGs in BRs signal pathways, one of each two *BAK1s*, *BKI1s* and *BZR1/2s* was inhibited and the other one in the three DEGs was induced, while two *BRI1s* and eight *CYCD3s* were all decreased. In the JA signal pathway, only one *COI1*, which is major node gene, was found and showed stable decreases. The expressions of nine *JAZs* (TIFY family) and three *MYC2s* increased significantly. In particular, most of the *JAZs*’ expressions increased at 3h in roots (Fig 7B).

In addition, 9 DEGs in GA signal transduction were detected. Among them, three *GID1s* were induced, especially the one *Gorai*.*008G007200* was in continuous expression at three time points in the two organs, whereas four *DELLA* proteins and two *PIFs* were totally inhibited under salt stress. Besides, the DEGs in cytokinin and IAA signal pathways showed similar trends with almost all genes repressed (Fig 7B).

### DEGs related with the oxidation-reduction process respond to salt stress

Salt stress induces reactive oxygen species (ROS), which leads to secondary oxidation stress, disturbs cellular redox homeostasis and damages cell components and structures. The alleviation of oxidative damage and increased resistance to salt stresses was often correlated with the balance between ROS producing and ROS scavenging [18]. In the present study, nine *RBOHs* (respiratory burst oxidase homologs), which take the responsibility for the accumulation of H_2_O_2_, were significantly up-regulated in both root and leaf tissues when suffering salt stress (Fig 7C; S7 Table). Simultaneously, ROS scavenging systems were markedly influenced, showing different expression patterns under salt treatment and the same models with the *G*. *davidsonii* transcriptome results [51]. In total, twenty-three members of peroxidase (*POD*) family were up-regulated in the two organs and most of them were up-regulated at 12h treatment in roots. In addition, two catalase (*CAT*) genes were up-regulated one in roots and one in leaves, respectively, and the superoxide dismutase (*SOD*) maintained no change. Most of the twenty-one glutathione S-transferase (*GST*) genes were significantly upregulated. Seven glutaredoxins (*GRXs*) genes and one Glutathione reductase (*GR*) were also upregulated, whereas a completely opposite expression pattern of two *PRX* genes with one upregulated in roots and another down-regulated in leaves under salt treatments was examined. These results were consistent with the high level of GSH (Fig 1C) and suggested that the enzymatic pathways of *POD*, *GST*, and *CAT* gene families play particularly important roles in protecting cotton against oxidative damage under salt stress.

### DEGs involved in ion homeostasis and Ca^2+^-related genes

The damage of ion homeostasis by excess Na^+^ will immediately activated Ca^2+^ signal pathway, Ca^2+^ acts as a secondary messenger in salt stress responses [68]. The cytosolic free Ca^2+^ concentration changes within seconds in plants subjected to salt stress. The salt overly sensitive (SOS) signaling pathway, a kind of Ca^2+^-related pathway, has been well documented at the cellular level, play the vital role in maintain ion homeostasis. In our study, six *SOS2* were upregulated in roots and leaves after post in salt stress (Fig 7D; S8 Table). Whereas, the level of *SOS3* remain unchanged, two *CBL10* genes were significant activated at three time points in roots. CBL10 (also known as SOS3-like calcium binding protein 8 SCaBP8), is more remarkable in roots in *Arabidopsis* than SOS3 [69]. Interesting, our result is consistent with the *G*. *davidsonii* transcriptome consequence that the significant activation of *CBL10* under salt stress in cotton [51]. Further, the Na^+^/H^+^ antiporter is an important protein in excluding Na^+^ at cellular level. In our data, *NHX7/SOS1* remain unchanged, while the vacuolar location gene *NHX2* (*Gorai*.*007G264500*) was found up-regulated in both tissues, especially in roots. Moreover, other genes involving the ion homeostasis were also found in our data. Such as the Na^+^ transporter gene *HKT6* (*Gorai*.*004G096400*; *Gorai*.*012G180500*) were down-regulated in roots. Such as the potassium transporters KUP (*Gorai*.*011G051700*; *Gorai*.*008G049100*; *Gorai*.*006G029100*; *Gorai*.*012G171200*; *Gorai*.*005G267900*), the plasma membrane ATPase (*Gorai*.*006G023600*; *Gorai*.*009G125200*) were also found to be up-regulated under salty stress.

In addition, four CIPK6 genes were upregulated in roots (S9 Table). As the previous studies reported that the tomato CIPK6 has been reported the interaction with CBL10 in transmission plant immunity signal in transgenic *N*. *benthamiana* [70], Furthermore, fourteen *CIPKs*, eighteen *CMLs* and eight Ca^2+^-ATPase genes showed significant activating by salt stress, and most of them up-regulated in roots, which revealed the important role of roots in perception and transmission salt signal.

### Examination of SOS-, ABA- and ROS-related genes by qRT-PCR

**Fig 8:**
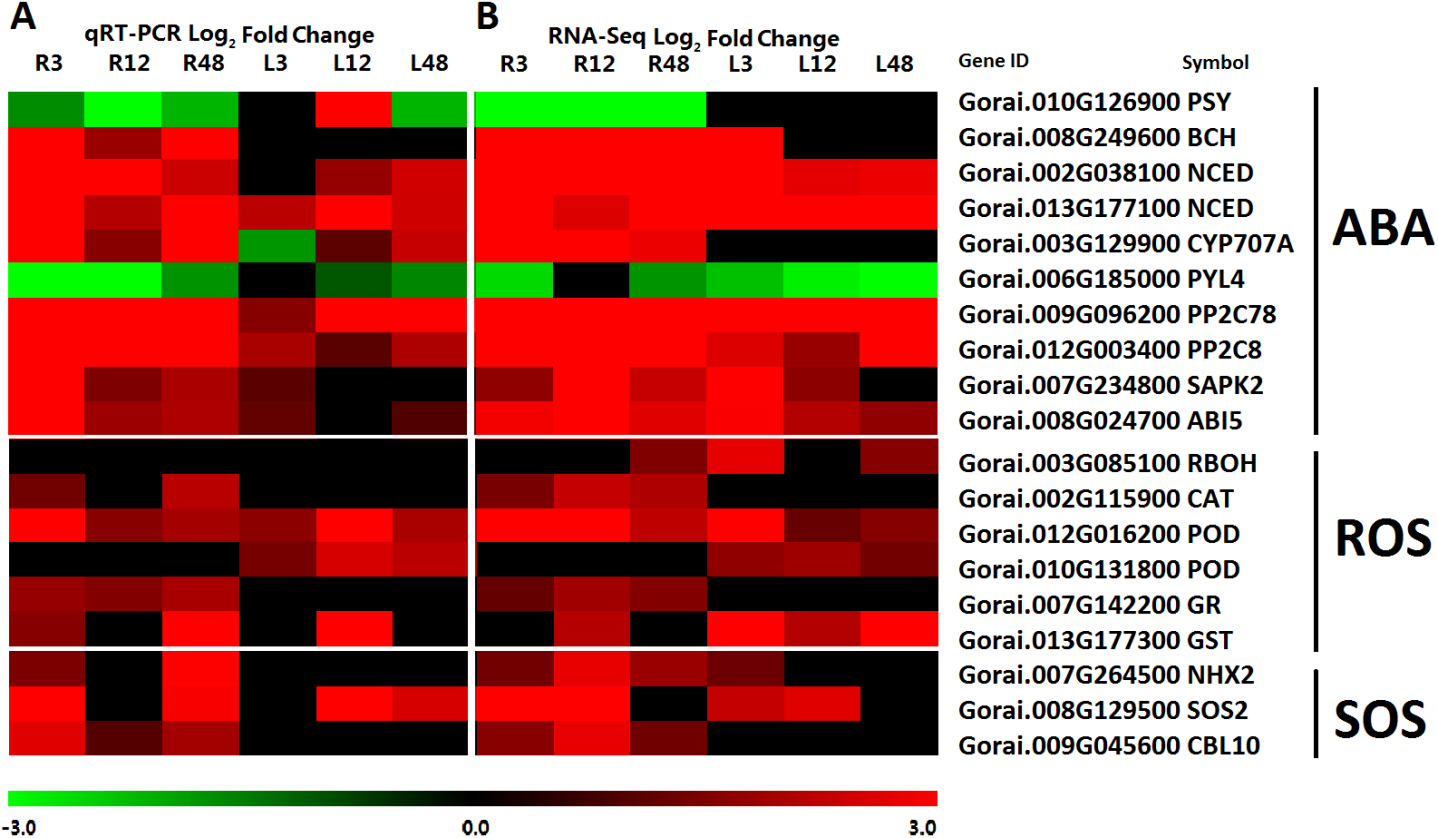
Heatmap analysis of the expression levels of ABA, ROS and SOS-related genes. **A:** A Heatmap by qRT-PCR Log_2_ Fold Change in roots and leaves of *G*. *klotzschianum*. B: A Heatmap by RNA-Seq Log_2_ Fold Change in roots and leaves of *G*. *klotzschianum*. The results were performed by the Fold Change ≥2.

To further verify the correctness of the analysis results, nineteen typical genes were selected and performed by RT-PCR, which contained ten ABA-related genes included five biosynthesis genes and five signal transduction genes, six ROS-related genes including one ROS-producing gene and five ROS-scavenging genes, three SOS-related genes (S1 Table-Further Examination). The qRT-PCR results revealed a good correlation with RNA-Seq data (Fig 8), which implied the reliability of our analysis results. All the chosen genes were five ABA biosynthesis related genes included one *PSY*, one *BCH*, two *NCEDs*, and one *CYP707A*, five ABA signal transduction genes included one *PYL*, two *PP2C*s, one *SnRK2*, and one *ABF*, six ROS-related genes included one *RBOH*, one CAT, two *POD*s, one *GR*, and one *GST*, and three SOS-related genes consisted of one *CBL10*, one *SOS2*, and one *NHX2*. These genes have been proved the important roles in response to salt stress by the previous studies [51, 62, 63, 67, 69], and the results in our data were in accordance with the previous, which showed the further support of the data veracity and the important roles of these genes in responding to salt stress in cotton.

### Identification of transcription factors responsive to salt stress in *G*. *klotzschianum*

Transcriptional modulation is vital aspects of the complex genetic and biochemical networks that plants use to respond to stress. TFs are master regulators that control gene clusters involved ABA-independent pathway and ABA-dependent pathway [71]. The increased level of ABA promotes the activity of downstream transcription factors to modulate the expression of various ABA-responsive genes [22], and stress-responsive transcription factors which have been extensively characterized in studies on plant stress tolerance [72]. Members of the *AP2/ERF*, *MYB*, *WRKY*, and *NAC* families were shown to regulate salt tolerance and many studies have shown that TFs could enhance salt tolerance through ABA signaling and the modulation of ROS production [73, 74]. In our data, 2348 TFs were discovered in total, 601 and 796 different expression TFs were identified in leaves and roots, respectively. These TFs were classified into 49 families (S10 Table). The most represented *G*. *klotzschianum* differentially expressed TFs families were *MYB*, *bHLH*, *ERF*, *NAC*, *bZIP*, and *WRKY*, which are known to mediate stress responses in plants, and the results were consistent with previous studies [75, 76]. The *NAC* family TFs, which is closely related with ABA signal pathway, showed significant up-regulated at the organs under salt stress. Moreover, six tissue-specific regulated TFs *YABBY* family proteins were also found to be altered in our data, as like the previous studies.

## Discussion

When salt stress occurs, two main factors are responsible for salt stress-induced inhibition of plant growth: osmotic stress and ionic stress [26]. As it is well known, the stress signal is first perceived by receptors, which results in generation of many secondary signal molecules, such as Ca^2+^, ROS and hormones [23, 77]. The signal molecules are then transduced into the nucleus, which leads to the activation of related genes to induce responses to salt stress [78]. In present study, RNA-seq was utilized to explore the time course of the response mechanism in cotton under salt stress. Various kinds of salt stress-related DEGs were found and the changes in expression under salt stress were consistent with previous reports. However, only three early time points were investigated in our study, we did concentrate on the ABA-, ROS-, and SOS-related signaling pathways induced by salt stress in the two organs.

In response to salt stress, the generation of ROS (Fig 1C c) may be achieved by activation of ROS-producing genes, such as *RBOHs* (Fig 7C). It has been demonstrated that ROS are produced by *RBOH* in a Ca^2+^-dependent manner [79]. However, plants have evolved a series of mechanisms to maintain the homeostasis of ROS [18]. In this study, we found that most of the *GPX*, *GST*, and *GRX* genes were expressed at higher levels under salt stress (Fig 7C). The enzymes encoded by these genes play important roles in the reaction of peroxide detoxification by catalyzing GSH to GSSG [80]. The involvement of this process was confirmed by the determination of GSH and GSSG contents (Fig 1C), which showed that the dramatically decrease of the GSH contents, and increase of the GSSG contents. The decrease in the AsA contents (Fig 1C) would also due to ROS scavenging. In addition, *POD* and *CAT* were found to be up-expressed, which was also consistent with previous report in which GhCAT1 acted as a ROS scavenger and participated in salt stress in cotton [36]. No *SOD* with different expression was found, suggesting that SODs may not be the main scavenger of ROS under short-term salt stress in cotton. These results suggest that GST, CAT, and POD may be the major scavengers of ROS in salt stress. Prior to their detoxication, genetic evidence suggests that ROS can also act as a signaling molecule in regulating diverse functions in plants [79]. However ROS function, not independently, but synergistically with other signaling pathways, can be regulated by transcription factors.

Hormones, in particular ABA, have been implicated in the stress signal transmission. ABA leads to activation of ion channels in guard cells and stomatal closure [72]. Ma et al. (2012) revealed that ABA would also induce H_2_O_2_ by activation of NADPH oxidases such as *AtRBOHD* and *AtRBOHF* in *Arabidopsis* [81]. ABA-induced H_2_O_2_ and then H_2_O_2_-activated Ca^2+^ channels are important mechanisms for ABA-induced stomatal closure [82]. Our transcriptomic data demonstrated that seven *RBOHs* were upregulated (Fig 7C) and the accumulation of H_2_O_2_ was maintained at a higher level (Fig 1C), which in turn triggers increased ABA biosynthesis, leading to further increases in H_2_O_2_ accumulation. Our results showed that many ABA biosynthetic genes (*PSY*, *BCH*, and *NCED*) were activated under salt stress (Fig 7A), but the ABA contents did not show a significant increase from initial stage (Fig 1C). This might be related to the high expression of four *ABAH4/CYP707A* genes (Fig 7A), which are key genes of ABA degradation to control ABA level [83]. In addition, ROS can affect the ABA signaling pathway by regulating *ABI1* and *ABI2*. In the presence of ABA, PYR/RCARs, as ABA receptors, interact with PP2Cs and inhibit phosphatase activity, allowing SnRK2 activation and phosphorylation of target proteins to control *ABI5* and *RBOH* gene expression [72, 84, 85]. Accordingly, we observed that five members of *RBOHA*, *B*, and *C* were significantly induced under salt stress (Fig 7C), which might be related to ABA signaling. Moreover, various other TFs belonging to several classes including *AP2/ERF*, *MYB*, *NAC*, and *HD-ZF* have been reported to be engaged in ABA-mediated gene expression. These results revealed the crosstalk of ABA and ROS in response to salt stress in cotton.

Once Na^+^ enters the cytosol, it can potentially be excluded (back to the soil) by Na^+^/H^+^ exchangers located in the plasma membrane, or sequestered into the vacuole by Na^+^/H^+^ exchangers (e.g. NHX proteins) located in the tonoplast [86–88]. The SOS signaling pathway was a well document CBL–CIPK pathway, which plays vital roles in the maintenance of ion homeostasis. Briefly, external stresses lead to the cytosolic free Ca^2+^ concentration changes within seconds, then SOS3 appeared to function as a primary calcium sensor by binding Ca^2+^ and activating SOS2, and the latest the SOS3–SOS2 complex stimulated the Na^+^/H^+^ exchanger activity of SOS [14], resulting in exclusion of excess Na^+^. In present study, seven antiporting ATPases were up-regulated. Whereas, the expression of *SOS3* and *SOS1* was unchanged, and several *SOS2*, two *CBL10*, and one tonoplast location Na^+^/H^+^ antiporter gene *NHX2* were up-regulated. The results were consistent with those in *G*. *davidsonii* at the early stage under salt stress [51]. As previous studies, increased calcium was perceived by two calcium-binding proteins, SOS3 and calcineurin B-like10 (CBL10, known as SOS3-like calcium-blinding protein8, SCaBP8), which interact with and activate the SOS2 serine/threonine protein kinase [69, 89, 90]. Further, CBL10 has been shown to be an alternative regulator of SOS2 [91, 92]. It was suggested that tonoplast Na^+^/H^+^ NHX antiporters are activated by AtCIPK24/SOS2 through the mechanism related to the AtCBL10 to sequester intracellular extra Na^+^ in the vacuole and AtCBL10–AtCIPK24/SOS2 was reported to regulate Na^+^ homeostasis in the vacuolar membrane in shoots and leaves [90, 93]. Therefore, we speculated that CBL10-SOS2 is paramount in the regulation of Na^+^ exclusion and cellular ion homeostasis at early stage in cotton. In addition, *HKT6*, which encodes a K^+^ transporter, was down-regulated at late stages under salt stress treatment in roots, likely leading to a decrease in Na^+^ and K^+^ uptake. Interestingly, all *KUPs* in roots and *KUP11* in leaves were up-regulated, likely leading to further K^+^ influx and maintaining the K^+^ balance of the cell. Together, these signaling pathways are involved in maintaining ion homeostasis to keep the plant away from ion toxicity.

**Fig 9:**
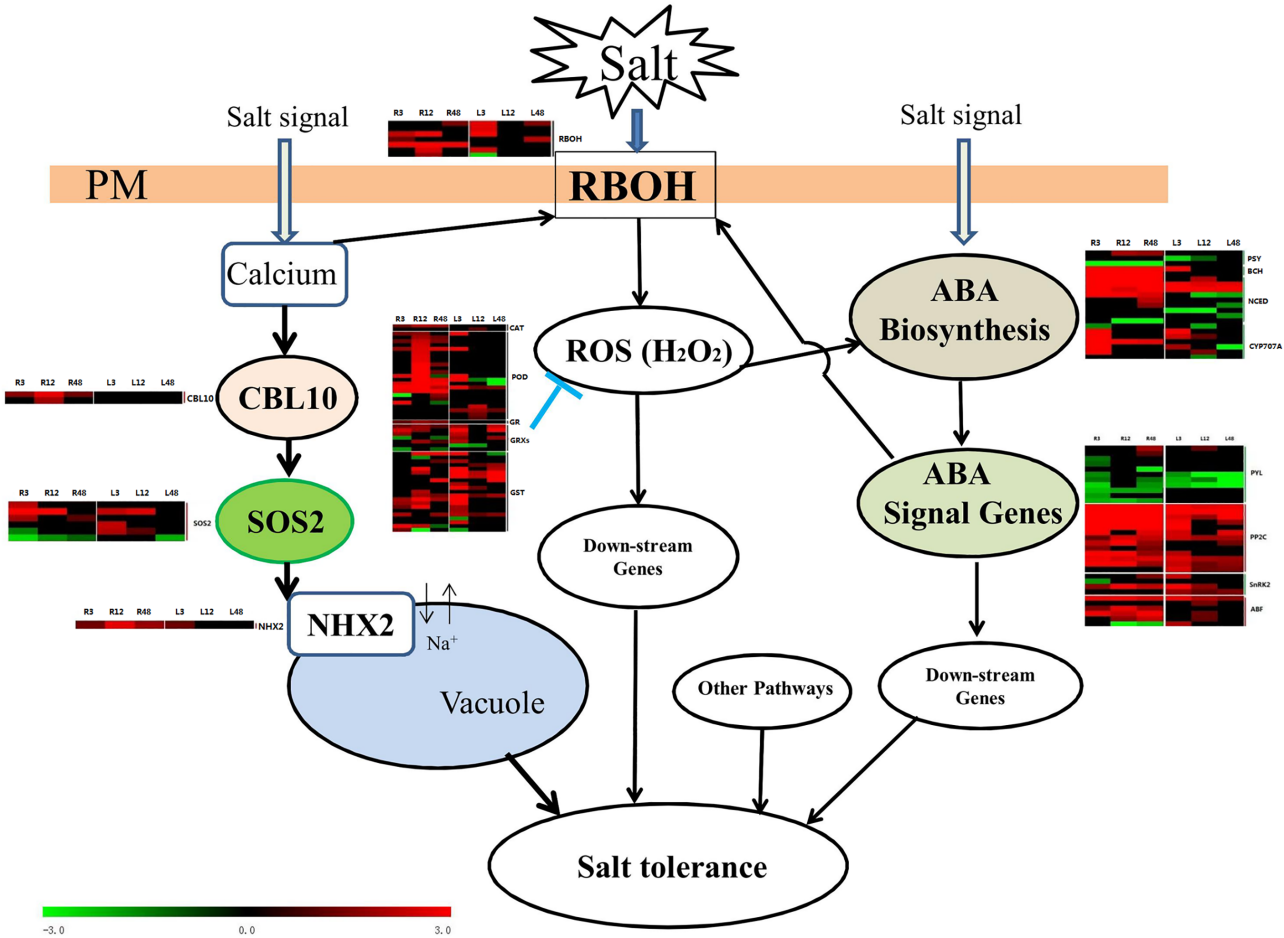
Hypothetical interaction network at early stage in response to salt stress in cotton. **Black arrow:** salt stress induced hormone biosynthesis and signal transduction, likewise, the SOS pathway in the early part of the treatment, which leads to H_2_O_2_ accumulation; **Blunt arrow:** negative regulation.

All of the signaling and metabolic pathways described could regulate gene expression involved in protecting plants from salt stress at different levels. Combined with previous studies, our results implied that there might be a connection among H_2_O_2_, ABA signal molecules transductions and SOS-related genes to mediate salinity stress in cotton (Fig 9). However, the response to salt stress is an all-dimensional and multitier event. We are still far from determining the exact nature of the full mechanisms of the response to salt stress in cotton.

In conclusion, we presented the first comprehensive transcriptome data from the wild diploid cotton species *G*. *klotzschianum*. The transcriptome revealed that 8,312 and 6,732 DEGs were discovered to be involved in salt stress tolerance in roots and leaves. Furthermore, the DEGs related to ROS, hormones and ion balance were significantly involved in the response to salt stress, especially at the initial stage of the salt treated two tissues, which may benefit cotton rapid response to cope with high salinity damage. Based on these findings, the crosstalk between ROS, ABA and Ca^2+^-related genes will be further elucidated in future cotton studies and also be very useful for salt resistance genetic improvement of the cultivated upland cotton.

## Supporting information

S1 Fig Heat map of 193 DEGs from fourteen samples. (TIF)

S1 Table Primer list of transcripts for real-time RT-PCR. (XLSX)

S2 Table Summary of differentially regulated genes during the time course. (XLSX)

S3 Table The common DEGs in roots and leaves. (XLSX)

S4 Table KEGG pathway enrichment of differentially expressed genes. (XLSX)

S5 Table Differentially expressed genes for hormone synthesis. (XLSX)

S6 Table Differentially expressed genes for hormone signal pathways. (XLS)

S7 Table Differentially expressed genes for ROS production and scavenging. (XLSX)

S8 Table Differentially expressed genes for ion balance. (XLSX)

S9 Table Differentially expressed genes for calcium-related. (XLSX)

S10 Table Differentially expressed TFs in roots and leaves. Data Section. RNA-Seq data in this study have been deposited at the NCBI under the accessions: SRR5500539.
(XLSX)
