# Supplementary figures and images for: Salt stress responsiveness of a wild cotton species (Gossypium klotzschianum) based on transcriptomic analysis
Source: PLoS One. 2017 May 26;12(5):e0178313. doi: 10.1371/journal.pone.0178313 (PMC5446155; doi:10.1371/journal.pone.0178313)

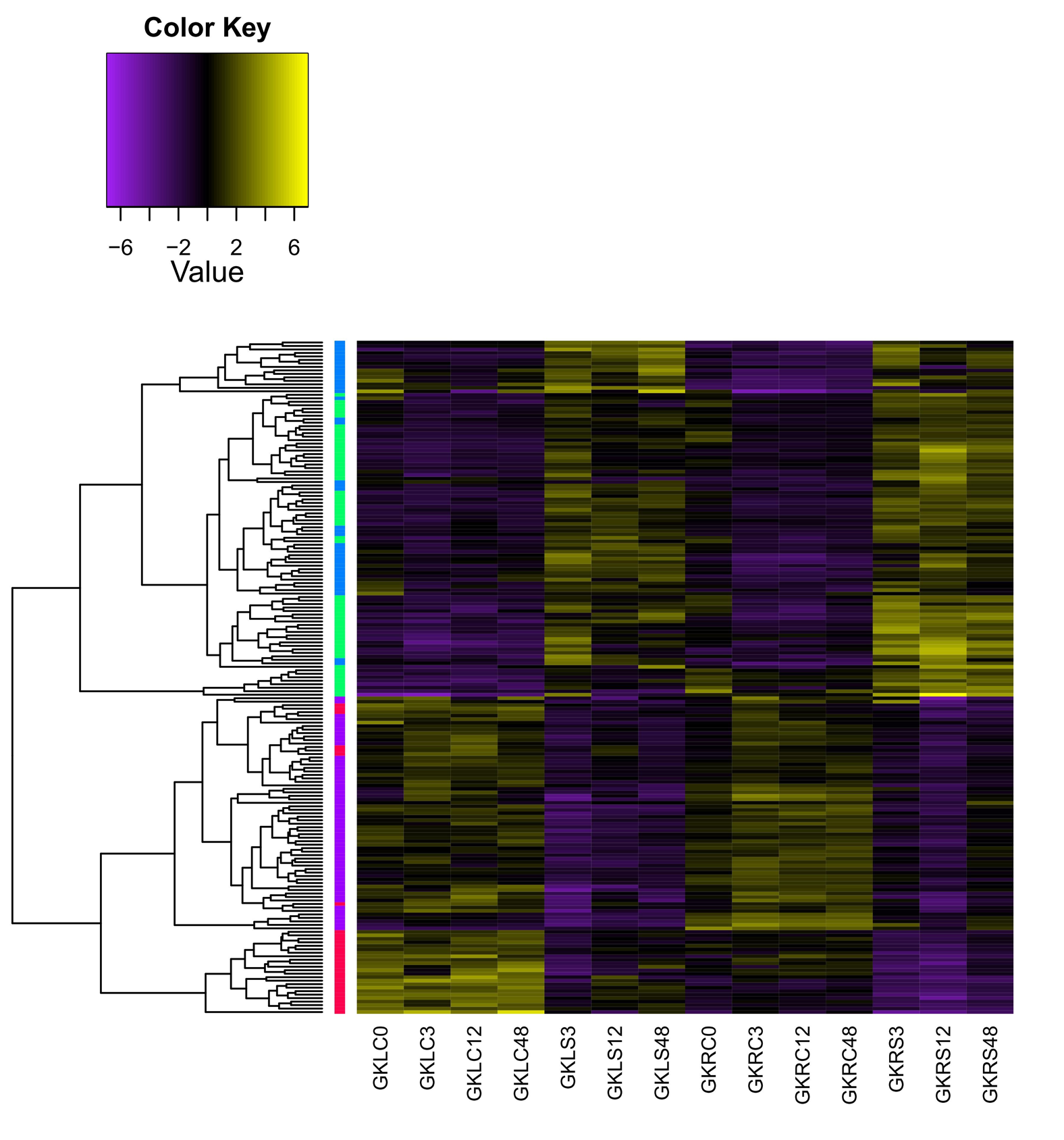

Supplement: S1 Fig — (TIF) [file pone.0178313.s001.tif]
